# Regulation of seed traits in soybean

**DOI:** 10.1007/s42994-023-00122-8

**Published:** 2023-11-27

**Authors:** Yang Hu, Yue Liu, Jun-Jie Wei, Wan-Ke Zhang, Shou-Yi Chen, Jin-Song Zhang

**Affiliations:** 1 State Key Lab of Plant Genomics, Institute of Genetics and Developmental Biology, Innovative Academy of Seed Design, Chinese Academy of Sciences, Beijing, 100101 China; 2 College of Advanced Agricultural Sciences, University of Chinese Academy of Sciences, Beijing, 100049 China

**Keywords:** Soybean, Seed weight and size, Oil, Protein

## Abstract

Soybean (*Glycine max*) is an essential economic crop that provides vegetative oil and protein for humans, worldwide. Increasing soybean yield as well as improving seed quality is of great importance. Seed weight/size, oil and protein content are the three major traits determining seed quality, and seed weight also influences soybean yield. In recent years, the availability of soybean omics data and the development of related techniques have paved the way for better research on soybean functional genomics, providing a comprehensive understanding of gene functions. This review summarizes the regulatory genes that influence seed size/weight, oil content and protein content in soybean. We also provided a general overview of the pleiotropic effect for the genes in controlling seed traits and environmental stresses. Ultimately, it is expected that this review will be beneficial in breeding improved traits in soybean.

## Introduction

Plants provide approximately 70% of the cooking oil and 50% of the dietary protein for humans, with soybean playing a significant role (Duan et al. 2023). Moreover, the natural nitrogen fixation provided by soybean roots reduces the use of fertilizers, making soybean a valuable crop for sustainable agricultural production. Therefore, it is of great importance to increase soybean yield and improve seed quality. Soybean yield is influenced by seed weight, which is usually positively associated with seed size (Li et al. 2018; Liu et al. 2020b; Hu et al. 2023a). Cultivated soybean seeds are composed of ~ 20% oil and ~ 40% protein, and both of these traits largely determine soybean seed quality (Lu et al. 2021a; Goettel et al. 2022). Seed size/weight, oil content and protein content are coordinately regulated by genetic factors and environmental signals (Duan et al. 2023). Increasing seed weight, oil content and protein content are important breeding goals. To date, a series of key factors controlling these traits have been identified in soybean, offering valuable targets for molecular breeding design.

Many crops have been domesticated from wild plant species (Doebley et al. 2006). A number of morphological and physiological changes between crops and their progenitors appeared during this process and this phenomenon is regarded as ‘domestication syndrome’ (Gaut et al. 2018). Cultivated soybean was domesticated from wild soybean (*Glycine soja*) in China approximately 6000–9000 years ago (Kim et al. 2010). Subsequently, cultivated soybean was introduced to East Asia and was later introduced to North America in the 1760s (Zhou et al. 2015). Soybean is now planted worldwide. During soybean domestication, through improvement and regional breeding, the genome diversity decreased significantly. Approximately 50% of the soybean genetic diversity was lost during the transition from wild soybean (*π* = 2.94 × 10^–3^) to soy landraces (*π* = 1.40 × 10^–3^) (Zhou et al. 2015). A small number of Asian landraces were introduced to North America and became the genetic base of the North American cultivars (Hyten et al. 2006). Understanding the soy genomic variation is therefore beneficial to future molecular breeding and soybean improvement.

Many soybean traits, such as stem growth habit, seed dormancy characteristics, flowering time and stress tolerance, changed significantly during the domestication process. Some domestication-related genes have been identified in recent years. *Dt1* encodes a homolog of phosphatidylethanolamine-binding protein, and the single-nucleotide substitutions of this gene, as a result of selective breeding, have influenced stem growth habits (Liu et al. 2010; Tian et al. 2010). The stay-green *G* gene was identified as a regulator of seed dormancy and has a conserved function in other species (Wang et al. 2018). GmPRR3b^H6^ represses the expression of *GmCCA1a*, which acts as a transcriptional activator for *J*, and overexpression of *GmPRR3b*^*H6*^ delays soybean flowering in natural, long-day (LD) and short-day (SD) conditions (Lu et al. 2017a; Li et al. 2020; Wang et al. 2020b). *HSFB2b* improves salt tolerance by activating the flavonoid biosynthesis pathway and shows evidence of selective breeding over the course of soybean domestication (Bian et al. 2020). In addition, between wild and cultivated soybean, there have been other noticeable changes in seed traits, such as the shift from small seeds to large seeds, from low to high oil content and from high to low protein content. Interestingly, these traits were often highly correlated in soybean seeds. The protein concentration often shows a negative correlation with seed yield and oil concentration (Bandillo et al. 2015; Zhang et al. 2022). Thus, gaining deeper insights into the regulatory networks and multiple functions of relevant genes is expected to be beneficial to soybean breeding practices.

The release of the soybean Williams 82 cultivar reference genome in 2010 opened the door for studies of soybean functional genomics (Schmutz et al. 2010). Then, the sequencing of the undomesticated soybean IT182932 provided detailed information on genetic variation between wild soybean and cultivated soybean (Kim et al. 2010). With the development of next-generation sequencing technology, more soybean reference genomes have been made available, including those of the cultivars Zhonghuang13, W05 and Jindou17 (Shen et al. 2018a, 2019; Xie et al. 2019; Yi et al. 2022). In addition, the pangenomes of wild and cultivated soybeans have also been constructed, launching a new era of evolutionary and functional genomics studies (Li et al. 2014; Liu et al. 2020c). The collection and analysis of 1298 transcriptome samples has provided a comprehensive view of soybean gene expression (Machado et al. 2020). Systematic analysis of the epigenome elucidated the relationship between DNA methylation and soybean genetic variation (Shen et al. 2018b). Other omics researches, such as proteomics and metabolomics, have also made progress in recent years (Komatsu et al. 2017; Silva et al. 2021), producing data that have facilitated the identification of soybean regulatory genes.

Here, we summarize the soybean functional genes and genetic pathways that regulate soybean seed size, oil content and protein content, with a focus on gene pleiotropism, and discuss the challenges and prospects for future soybean studies.

## Control of seed weight and size in soybean

Seed weight demonstrates broad variation in the plant kingdom but exhibits narrow variation within species (Westoby et al. 1996). Seed weight and size play important roles not only in plant fitness and adaptation, but also in yield determination. On the one hand, seed weight is correlated with a series of plant adaptabilities to the environment, such as dispersal mode, plant height, leaf area and stress tolerance (Westoby et al. 1996; Moles et al. 2005). On the other hand, seed weight is correlated with seed number, and both influence seed yield (Liu et al. 2020b).

In angiosperms, seed development is an essential process in the life cycle. This process occurs through double fertilization, which is a unique characteristic of flowering plants. During double fertilization, one sperm cell fuses with the egg cell to generate the diploid zygote, and the diploid central cell is fertilized by the other sperm cell to form the triploid endosperm (Goldberg et al. 1989). The fertilized zygote develops into an embryo during embryogenesis, and the endosperm facilitates seed germination by delivering nutrients to the embryo (Chaudhury et al. 2001). The seed coat, which comes from sporophytic integuments, is also an important component of mature seeds (Li et al. 2019). It offers support and protection for the embryo (Figueiredo and Kohler 2014). Thus, seed development is determined coordinately by zygotic and maternal tissues in addition to environmental signals (Li and Li 2015). In recent years, several signaling pathways that influence seed size, including the ubiquitin–proteasome pathway, G-protein signaling, mitogen-activated protein kinase signaling, phytohormone pathway and transcriptional regulator pathway, have been identified (Li et al. 2019).

In rice, the spikelet hull encloses the embryo and endosperm and determines the final grain size, predominantly by setting the storage capacity and limiting grain growth during the rice development process (Li et al. 2018). The volume of the spikelet hull is regulated by the maternal genotype, and the grain weight is determined maternally under this condition (Li et al. 2019). The rice otubain-like protease WTG1 and RING-type protein GW2 negatively regulate grain weight by affecting spikelet hulls (Song et al. 2007; Huang et al. 2017). OsMKKK10-OsMKK4-OsMAPK6 acts in a module to enhance grain size by promoting cell proliferation (Duan et al. 2014; Liu et al. 2015; Xu et al. 2018). Soybean seed development can be divided into three stages, namely, morphogenesis, maturation and desiccation (Le et al. 2007; Nguyen et al. 2016). The process is accompanied by pod development, which provides a space for the seeds. The soybean endosperm is absorbed by the embryo and disappears during seed development (Goldberg et al. 1989). In mature soybean seeds, cotyledons occupy most parts of the embryo and influence seed size. The seed coat surrounds the embryo and provides nutrition to support seed development (Déjardin et al. 1997). Hence, soybean seed weight/size is controlled coordinately by maternal and zygotic tissues. Some regulatory genes controlling seed size have been identified by studying pod development, such as *SoyWRKY15a*, while more genes, such as *GA20OX*, *GmCYP78A5* and *GmJAZ3*, have been found by analyzing RNA-seq data from various seed developmental stages (Lu et al. 2016; Du et al. 2017; Gu et al. 2017; Hu et al. 2023a).

In the past decade, great progress was made in elucidating soybean seed size regulation (Table 1). Overexpression of *GmCYP78A72* was shown to promote seed size, both in *Arabidopsis* and in soybean, whereas silencing of this *P450* gene in soybean does not cause obvious phenotypic changes (Zhao et al. 2016). Further silencing of the other two *GmCYP78A* genes resulted in small soybean seeds, indicating the functional redundancy of this gene family. Upregulation of *GmCYP78A5* expression, another cytochrome P450 family gene, also increased seed size and weight (Du et al. 2017). *GmCIF1* encodes a cell wall invertase inhibitor, and RNAi of *GmCIF1* increases cell wall invertase activities, leading to heavier seeds (Tang et al. 2017). *GmSWEET10a* is located in a selective sweep region and regulates seed size by transporting sucrose and hexose (Wang et al. 2020a). Its homolog *GmSWEET10b* exhibits a similar function in increasing seed weight. Downregulation of *GmBS1* and *GmBS2 *results in significantly increased size and weight of plant organs, such as seeds, pods and leaves (Ge et al. 2016). The expression of the *GRF5*, *GIF1*, *CYCD3;3* and *HISTONE4* genes was also shown to be increased in these *GmBS*-silenced soybean plants compared with the Williams 82 plants. Soybean GmFULa acts as a transcription factor and promotes biomass accumulation and 100-seed weight (Yue et al. 2021). Through coexpression network analysis, *GmJAZ3* was identified as a seed development regulator, and the GmJAZ3-GmRR18a-GmMYC2a-GmCKXs module promotes seed size by orchestrating jasmonate and cytokinin signaling (Hu et al. 2023a). The *GmJAZ3* Hap3 promoter was selected from wild soybeans and has been fixed in cultivars. The *GmCOL2b*-transgenic soybean was found to produce larger seeds than the wild type (WT) under both SD and LD conditions, whereas knockout of *GmCOL2b* produced smaller seeds only under SD conditions (Yu et al. 2023). Compared with mock-inoculated and vector-infected soybeans, soybeans with three *GmFAD3* genes (*GmFAD3A*, *GmFAD3B* and *GmFAD3C*) silenced produced both larger and heavier seeds (Singh et al. 2011). In another study, suppressing the expression of four TAG lipases did not alter seed number but did increase seed weight, thereby promoting seed yield in soybean (Kanai et al. 2019). Compared with the WT, overexpression of *GmOLEO1* also decreased seed weight, but increased seed pod numbers, which ultimately enhanced seed yield (Zhang et al. 2019). GmPLATZ was identified through analyzing the transcriptomes of developing seeds, and was shown to directly activate the expression of cyclin genes and *GmGA20OX* to enhance soybean seed size and weight (Hu et al. 2023b).

**Table 1:** Regulatory genes for soybean seed traits

Gene name	Accession number	Annotation	Functions in seed traits			References
			Seed weight/size	Oil content	Protein content	
*GmFAD3A*	Glyma.03G056700	Omega-3 fatty acid desaturase	Decreased	N.A.	N.A.	Singh et al. (2011)
*GmFAD3B*	Glyma.07G151300					
*GmFAD3C*	Glyma.11G174100					
*GmBS1*	Glyma.10G244400	TIFY family protein	Decreased	N.A.	N.A.	Ge et al. (2016)
*GmBS2*	N.A.					
*GmCYP78A57*	Glyma.02G119600	Cytochrome P450 family	Increased	N.A.	N.A.	Zhao et al. (2016)
*GmCYP78A70*	Glyma.01G061100					
*GmCYP78A72*	Glyma.19G240800					
*GmCYP78A5*	Glyma.05G019200	Cytochrome P450 family	Increased	N.A.	N.A.	Du et al. (2017)
*GmZF351*	Glyma.06G290100	Zinc finger transcription factor	N.A.	Increased	N.A.	Li et al. (2017)
*GmCIF1*	Glyma.17G036300	Cell wall invertase inhibitor	Decreased	N.A.	Decreased	Tang et al. (2017)
*GmWRI1a*	Glyma.15G221600	AP2/EREBP transcription factor	N.A.	Increased	N.A.	Chen et al. (2018)
						Wang et al. (2022b)
*B1*	Glyma.13G241700	Transmembrane transporter-like protein	N.A.	Decreased	N.A.	Zhang et al. (2018a)
*GmSDP1-1*	Glyma.02G190000	TAG lipase	Decreased	Decreased	Increased	Kanai et al. (2019)
*GmSDP1-2*	N.A.					
*GmSDP1-3*	N.A.					
*GmSDP1-4*	N.A.					
*GmOLEO1*	Glyma.20G196600	Oleosin protein	Decreased	Increased	Decreased	Zhang et al. (2019)
*GmWRI1b*	Glyma.08G227700	AP2/EREBP transcription factor	N.A.	Increased	N.A.	Guo et al. (2020)
*GmPDAT*	Glyma.13G108100	Phospholipid:diacylglycerol acyltransferase	Increased	Increased	N.A.	Liu et al. (2020a)
*GmKIX8-1*	Glyma.17G112800	KIX domain-containing protein	Decreased	N.A.	N.A.	Nguyen et al. (2020)
*GmNAP1*	Glyma.20G019300	NCK-associated protein	Increased	N.A.	N.A.	Tang et al. (2020)
*GmSWEET10a*	Glyma.15G049200	Sugar transporter	Increased	Increased	Decreased	Wang et al. (2020a)
*GmSWEET10b*	Glyma.08G183500					
*GmDGAT2A*	Glyma.09G195400	Diacylglycerol acyltransferase	N.A.	Increased	N.A.	Jing et al. (2021)
*GmZF392*	Glyma.12G205700	Zinc finger transcription factor	N.A.	Increased	N.A.	Lu et al. (2021a)
*GmNFYA*	Glyma.02G303800	Nuclear transcription factor Y subunit A				
*GmDGAT1-2*	Glyma.17G053300	Diacylglycerol acyltransferase	N.A.	Increased	N.A.	Xu et al. (2021)
*GmFULa*	Glyma.06G205800	MADS-box transcription factor	Increased	N.A.	N.A.	Yue et al. (2021)
*GmGPDHp1*	Glyma.02G218700	Glycerol‐3‐phosphate dehydrogenase	N.A.	Increased	N.A.	Zhao et al. (2021)
*GmST05/GmMFT*	Glyma.05G244100	Phosphatidylethanolamine-binding protein	Increased	Increased	Decreased	Duan et al. (2022)
						Cai et al. (2023)
*POWR1*_*-TE*_	Glyma.20G085100	CCT domain-containing protein	Decreased	Decreased	Increased	Goettel et al. (2022)
*GmGA3ox1*	Glyma.07G033800	Gibberellin 3b-hydroxylase	Increased	N.A.	N.A.	Hu et al. (2022)
*ST1*	Glyma.08G109100	UDP-glucose 4-epimerase	Increased	N.A.	N.A.	Li et al. (2022)
*Dt2*	Glyma.18G273600	MADS-box transcription factor	Decreased	N.A.	N.A.	Liang et al. (2022)
*PG031*	Glyma.06G207300	Polygalacturonase protein	Decreased	N.A.	N.A.	Wang et al. (2022a)
*GmSSS1*	Glyma.19G196000	SPINDLY homolog protein	Increased	N.A.	N.A.	Zhu et al. (2022)
*GmSW16.1*	Glyma.16G198300	LIM domain-containing protein	Increased	N.A.	N.A.	Chen et al. (2023)
*GmJAZ3*	Glyma.09G123600	TIFY family protein	Increased	Decreased	Increased	Hu et al. (2023a)
*GmFtsH25*	Glyma.18G065600	Filamentation temperature‐sensitive protein H protease	Increased	N.A.	N.A.	Wang et al. (2023)
*GmCOL2b*	Glyma.19G039000	Zinc finger protein	Increased	N.A.	N.A.	Yu et al. (2023)
*hsw*	Glyma.11G095200	β-1, 3-glucosidase	Increased	N.A.	N.A.	Wei et al. (2023a)
*GmPLATZ*	Glyma.13G147800	Zinc finger transcription factor	Increased	N.A.	N.A.	Hu et al. (2023b)
*GmGA20OX*	Glyma.07G081700	Gibberellin 20-oxidase				

Owing to the development of sequencing technology and the improvement of the reference genome, a growing number of functional soybean genes have been identified through forward genetic methods. The *PP2C-1* allele from wild soybean promotes seed size by enhancing cell size, and PP2C-1 was shown to influence BR signaling by interacting with and dephosphorylating GmBZR1 (Lu et al. 2017b). *GmPDAT* has also been identified as a seed size regulator, by genome-wide association studies (GWAS), and subsequent research revealed that overexpression of this gene increased seed size, whereas knockdown of the gene by RNAi decreased seed size (Liu et al. 2020a). Compared with the WT, the K83 mutant was shown to have larger and heavier seeds, with *GmKIX8-1* being the causative gene producing this mutant (Nguyen et al. 2020). The mutant plants created by CRISPR have shown elevated expression of *GmCYCD3;1–10* and increased cell proliferation. The *Gmdtm1-1* and *Gmdtm1-2* mutants have shorter trichomes, reduced plant height and smaller seeds, and here *GmNAP1* was the causative genetic locus responsible for these variations (Tang et al. 2020). 

*GmST05* was identified by GWAS from over 1800 soybean accessions and was shown to positively regulate seed thickness, length and width (Duan et al. 2022). Promoter variations of *GmST05* could influence the expression of this gene, in pods and seeds, to alter final seed weight. Through GWAS and quantitative trait locus (QTL) analyses, the *GmMFT* gene was also identified and named (Cai et al. 2023). Overexpression of *GmMFT* increased seed weight, whereas its mutants exhibited decreased seed weight. A 321-bp transposable element insertion in the CCT domain of *POWR1* was also shown to influence POWR1 protein localization and to increase soybean seed weight (Goettel et al. 2022). By analyzing the sequencing data of 184 RILs developed from the KF No. 1 and NN 1138-2 and from 211 soybean accessions, *GmGA3ox1* was identified as a seed weight regulator (Hu et al. 2022). Knockout of *GmGA3ox1* upregulated the net photosynthesis rate and downregulated seed weight by decreasing the GA content. *ST1* is a domesticated gene that controls seed size by regulating pectin biosynthesis (Li et al. 2022). *Dt2* encodes a MADS-box transcription factor, and compared with the DN50 control, *Dt2* knockout mutant plants exhibit more branches, as well as larger and heavier seeds (Liang et al. 2022). 

In another study, the expression of *PG031* was induced in the seed coat and radical during germination, and these *PG031*-overexpressing transgenic lines produced smaller and lighter seeds than the WT (Wang et al. 2022a). The *sss1* mutant also had altered 100-seed weight by influencing both cell area and cell number (Zhu et al. 2022). This mutant was mapped to the *GmSSS1* gene, which encodes a putative O-GlcNAc transferase that regulates the expression of the *GmGAI1* and *GmBZR1* genes. *SW16.1* was identified from a population derived from NN 1138–2 and the wild soybean chromosome segment substitution line CSSL3068 and encodes a LIM domain-containing protein (Chen et al. 2023). Interestingly, the *GsSW16.1* allele from wild soybean negatively regulates seed weight, whereas the *GmSW16.1* allele from cultivated soybean positively regulates seed weight. Combining the results from linkage analysis and GWAS, *GmFtsH25* was identified as a regulator of photosynthesis (Wang et al. 2023). The overexpression of this filamentation temperature-sensitive protein H protease promotes soybean branch number, pod number and 100-seed weight. Transgenic soybean plants overexpressing *hsw* were able to produce larger and heavier seeds (Wei et al. 2023a).

## Control of seed oil content in soybean

Soybean fatty acids (FAs) accumulate over a relatively short period (4–6 weeks) and are generally stored in the cotyledons (Nguyen et al. 2016). Plant FAs in seeds are mainly stored in the form of triacylglycerols (TAGs), and their biosynthesis requires a carbohydrate flux, such as in the form of sucrose from photoautotrophic tissues (Song et al. 2013). In *Arabidopsis*, disruption of *AtSUC5*, a sucrose transporter gene, resulted in a decreased FA concentration in seeds (Baud et al. 2005). Lipid accumulation was initiated from the de novo synthesis of FAs from acetyl-CoA, which was generated from pyruvate during glycolysis. Acetyl-CoA carboxylase is the rate-limiting enzyme of FA synthesis and catalyzes the formation of malonyl-CoA from acetyl-CoA in an ATP-dependent step (Harwood 1988; Sasaki and Nagano 2004). The FA synthase complex then catalyzes elongation reactions in plastids (Ohlrogge and Browse 1995; Rawsthorne 2002). FA products are then exported to the endoplasmic reticulum to form TAGs. Many studies have explored this process, and the ‘Kennedy pathway’ has been well studied. In this pathway, FA products are catalyzed by glycerol-3-phosphate acyltransferase, lysophosphatidic acid acyltransferase, phosphatidic acid phosphatase and diacylglycerol acyltransferase (Kennedy 1961; Settlage et al. 1998). These synthesized TAGs are stored in oil bodies, which are surrounded by a phospholipid monolayer embedded with abundant proteins (Tzen et al. 1993; Napier et al. 1996). Most of these proteins were shown to be oleosins, and caleosins, and steroleosins were also identified (Jolivet et al. 2004).

Soybean oil is mainly composed of five FAs, namely, palmitic acid (16:0), stearic acid (18:0), oleic acid (18:1), linoleic acid (18:2) and linolenic acid (18:3). Palmitic acid and stearic acid are saturated FAs that account for approximately 17% of the total FAs in soybean (Demorest et al. 2016). *FAT* encodes an acyl carrier protein thioesterase, and knockout of *GmFATB1a* or *GmFATB1b* reduced the content of two saturated FAs (Ma et al. 2021). The combination of mutations within *GmFATB1a* and *GmFATB1b* can result in a more significant decrease in palmitic acid and stearic acid in soybean. Compared with the Forest control, the mutation of soybean *GmSACPD-A*, *GmSACPD-B,* and *GmSACPD-D,* was shown to increase the stearic acid content (Lakhssassi et al. 2020). FAD2 catalyzes the conversion of oleic acid to linoleic acid, and two copies (FAD2-1 and FAD2-2) exist in soybean (Okuley et al. 1994; Lakhssassi et al. 2017). 

The targeted mutagenesis of *GmFAD2-1A* and *GmFAD2-1B, *using TALENs, could dramatically influence the FA profile (Haun et al. 2014). Specifically, homozygous mutants had increased oleic acid content, but decreased linoleic acid content. The mutation of *GmFAD2-1A* and *GmFAD2-2A*, using CRISPR–Cas9, also elevated the oleic acid content, but significantly reduced the linoleic acid content (Wu et al. 2020). The double mutant of these two *GmFADs* produced a more marked effect than the single mutant. The WT contained 17.3% oleic acid and 59.5% linoleic acid, whereas the highest (65.9%) and the lowest (16.1%) levels of linoleic acid were detected in the *GmFAD2-2* mutants (Al Amin et al. 2019). FAD3 is the key enzyme that catalyzes the formation of linolenic acid from linoleic acid (Yadav et al. 1993; Demorest et al. 2016). Silencing of three soybean *FAD3* genes positively regulated the linoleic acid content, but negatively regulated the linolenic acid content (Flores et al. 2008). Compared with the *fad2-1a fad2-1b* soybean, the *fad2-1a fad2-1b fad3a* mutant plants produced lower linolenic acid (Demorest et al. 2016). Similarly, the three null mutant alleles (for *GmFAD3-1a*, *GmFAD3-1b* and *GmFAD3-2a*) were also shown to produce lower linolenic acid levels than the double mutants (for *GmFAD3-1a* and *GmFAD3-1b*) (Hoshino et al. 2014). In addition, knockout of two *GmPDCTs*, by CRISPR–Cas9, could elevate monounsaturated FAs but reduce polyunsaturated FAs in soybean seeds without influencing plant growth (Li et al. 2023).

Manipulation of related enzymes in lipid metabolism provided novel insight into the regulation of total oil content (Table 1). SDP1 catalyzes TAG degradation for postgermination growth, and a combination of mutations within *SDP1* genes contributes to FA accumulation in soybean seeds (Graham 2008; Kanai et al. 2019). PDAT is involved in plant TAG assembly and plays important roles in seed development (Zhang et al. 2009; Fan et al. 2013). In Williams 82, overexpression of *GmPDAT* positively regulated oil content, whereas RNAi of *GmPDAT* could negatively regulate oil content (Liu et al. 2020a). DGAT is responsible for the final step of the ‘Kennedy pathway’ and transfers an acyl group from acyl-CoA to diacylglycerol (Bates et al. 2013; Jing et al. 2021). Both *GmDGAT2A-* and *GmDGAT1-2*-overexpressing soybean plants were shown to exhibit increased total oil content (Jing et al. 2021; Xu et al. 2021). GmGPDHp1 contributes to the formation of glycerol-3-phosphate, and more oil bodies accumulate in *GmGPDHp1*-transgenic soybean seeds than in WT seeds (Zhao et al. 2021).

To date, several studies have revealed the regulatory genes involved in controlling total oil content in soybean, and these transcription regulators play essential roles in this process (Table 1). Overexpression of *GmWRI1a*, an AP2/EREBP family transcription factor, under the control of the *35S* promoter significantly enhance seed oil content (Wang et al. 2022b). A transcriptome analysis indicated that the carbohydrate and lipid metabolism pathways were enriched. Another study showed that overexpression of *GmWRI1a*, driven by a seed-specific promoter, also increased FA content (Chen et al. 2018). *GmWRI1a* participates in various steps of lipid accumulation by binding to the AW-box of regulatory genes. Upregulating the expression of the *GmWRI1b* gene, the homolog of *GmWRI1a*, results in an increase in oil and seed production (Guo et al. 2020). The B3 domain transcription factor, *GmLEC2*, was shown to enhance the TAG content and to activate the expression of lipid biosynthesis genes (Manan et al. 2017). *GmLEC1* is a central regulator of seed development and is also involved in lipid storage (Jo et al. 2020).

While most of the above genes from soybean that have been studied represent homologs from *Arabidopsis*, several transcription factor genes were first identified in soybean. *GmDof4* and *GmDof11* positively regulate seed FAs by activating the *accD* and long-chain acyl-CoA synthetase genes, respectively (Wang et al. 2007). *GmbZIP123* has a higher abundance during the lipid accumulation stage, and enhances oil content by promoting the expression of the *SUC* and *cwINV* genes (Song et al. 2013). GL2 downregulates oil content by influencing *PLDα1* expression, directly, and the MYB transcription factor GmMYB73 associates with GL3 and EGL3 to suppress *GL2* expression (Liu et al. 2014). Overexpression of *GmDREBL* promoted plant lipid accumulation and activated *WRI1* expression through binding to its promoter (Zhang et al. 2016). 

By establishing cultivar-specific gene coexpression networks, *GmNFYA* was identified as a hub gene, and its overexpression increased FA content (Lu et al. 2016). *GmZF351* was also identified from cultivar-specific gene coexpression networks and increased oil content in transgenic *Arabidopsis* and soybean (Li et al. 2017). It was shown to promote *WRI1* and *WRI*-regulated gene expression, directly, and has been selected for over the course of soybean domestication. GmZF392 interacts with GmZF351 to synergistically activate gene expression in the oil biosynthesis pathway, enhancing the oil content in soybean (Lu et al. 2021a). GmNFYA promotes the expression of the *GmZF351* and *GmZF392* genes to regulate soybean seed oil. While affecting seed size, the transcriptional repressor GmJAZ3 decreased seed oil content, likely by influencing sugar transportation (Hu et al. 2023a).

**Fig. 1:**
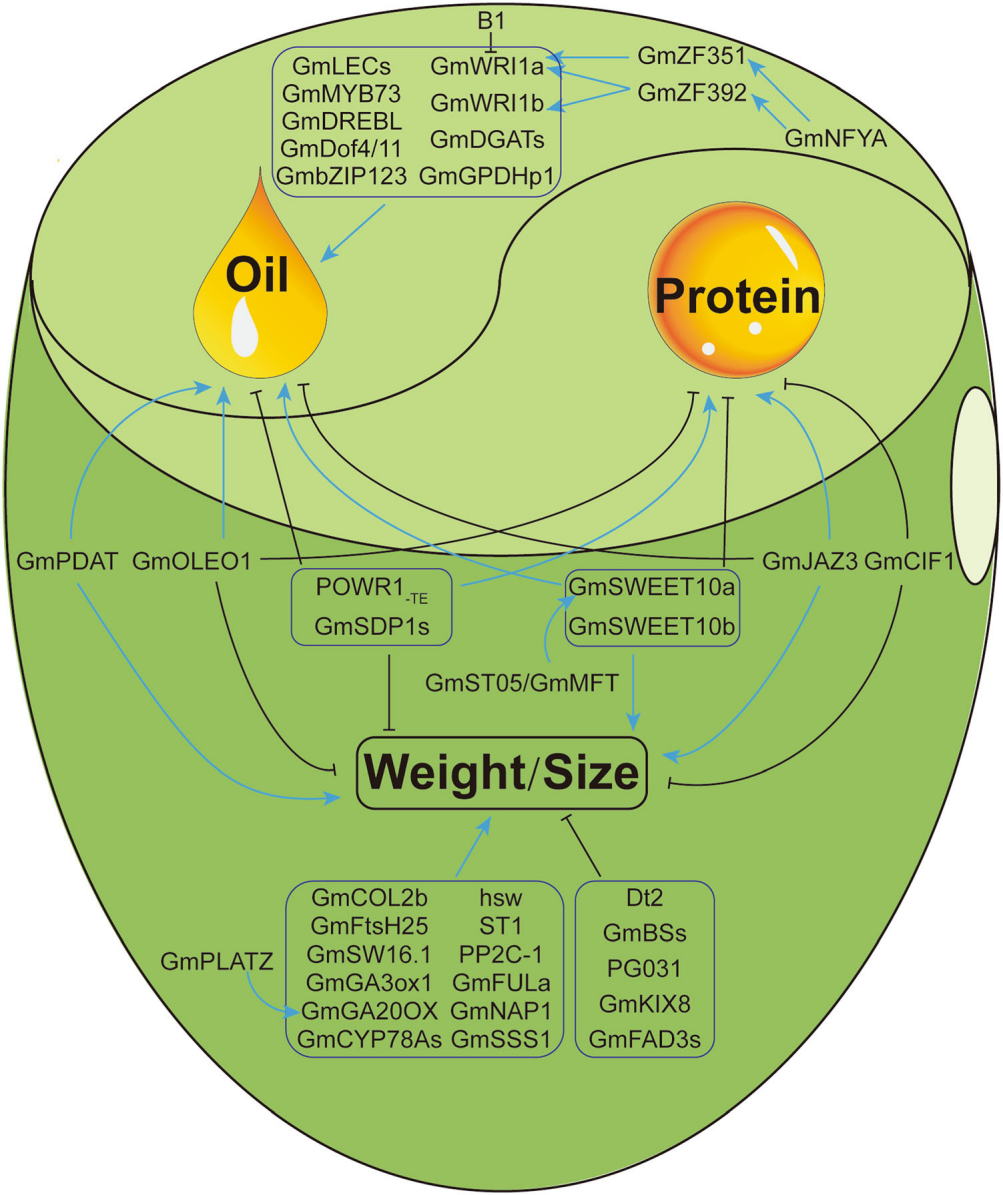
Schematic overview of seed trait control in soybean. The blue arrows indicate activation, and the black T-ended arrows indicate inhibition

Other genes were also shown to be involved in oil regulation. For example, *B1* can suppress expression of the *GmWRI1a*, *GmLEC1a*, *GmLEC1b* and *GmABI3b* genes in the soybean pod endocarp, which leads to a reduction in oil content (Zhang et al. 2018a). Through QTL and GWAS analyses for oil content, *GmOLEO1* was also identified (Zhang et al. 2019) and GmOLEO1 was localized in oil bodies and promoted lipid content in transgenic soybean seeds. Both *GmSWEET10a* and *GmSWEET10b* were shown to enhance FA content by influencing seed sugar allocation (Wang et al. 2020a). The expression of *GmSWEET39* is positively correlated with oil content in soybean (Miao et al. 2020). *GmST05*/*GmMFT* enhances seed oil content by activating the expression of *GmSWEET* genes (Duan et al. 2022; Cai et al. 2023). POWR1_-TE_ can decrease lipid content by affecting a series of oil metabolism-related genes, such as *OLEO1*, *WRI1a* and *ABI5* (Goettel et al. 2022). Finally, it is worth noting that many genes pleiotropically affect seed weight and lipid content (Fig. 1).

## Multiple functions of genes in seed trait regulation

Soybean protein accumulates mainly in the maturation stage of seed development (Le et al. 2007). Storage protein in soybean seeds is composed of 2S, 7S, 11S and 15S proteins, according to their sedimentation properties (Kinsella 1979). β-Conglycinin (7S) and glycinin (11S) are the major components of soybean protein, accounting for 70%-80% of total protein (Singh et al. 2015). These two protein contents are negatively correlated in mature seeds. Glycinin consists of six subunits, each of which is composed of an acidic chain and a basic chain, which are linked by disulfide bonds (Catsimpoolas 1969; Staswick et al. 1981). The subunits are divided into two groups based on physical properties, such as molecular weight and methionine (Nielsen 1985). Group I contains the G1, G2 and G3 subunits, and group II includes the G4 and G5 subunits. Sequence similarities range from 80 to 90% within the groups, but there is less than 50% similarity between the two groups (Nielsen et al. 1989). Other genes encoding glycinin subunits have also been identified in soybean (Beilinson et al. 2002). β-Conglycinin consists of three subunits: *α*, *α’* and *β*, with isoelectric points of 4.90, 5.18 and 5.66–6.00, respectively (Thanh and Shibasaki 1977; Tsubokura et al. 2012). At least 15 genes have been identified as encoding *β*-conglycinin and have been shown to be clustered into two groups based on mRNA length (Harada et al. 1989). The 11S proteins may only serve as storage nutrients, whereas the 7S proteins have shown potential roles in regulating plant development (Komatsu and Hirano 1991; Singh et al. 2015). Although the understanding of the storage protein species is relatively clear, the regulation of these components requires further investigation.

In soybean, many genes pleiotropically regulate seed traits, and recent studies have mainly focused on seed weight/size, oil content and protein content (Fig. 1). Some regulatory genes were shown to inversely influence FA and protein accumulation. *GmOLEO1*, *GmSWEET10s* and *GmST05*/*GmMFT* positively regulate oil content, but negatively regulate protein content (Zhang et al. 2019; Wang et al. 2020a; Duan et al. 2022; Cai et al. 2023). In contrast, *GmSDP1s*, *POWR1*_*-TE*_ and *GmJAZ3* decrease the oil content, but increase the protein content in mature seeds (Kanai et al. 2019; Goettel et al. 2022; Hu et al. 2023a). Carbon resources are fundamental substances for both oil and protein synthesis. Oil bodies and protein bodies exist simultaneously in soybean cells (Nguyen et al. 2016). Thus, these two nutrients influence each other by competing for a relatively constant precursor and space. Considering that both oil and protein are components of soybean seeds, alteration of the content of a single nutrient would affect the total seed weight. Many genes controlling oil and/or protein content have been shown to influence seed weight (Fig. 1). It is interesting to note that soybean regulatory genes often affect seed oil content and seed weight positively, while influencing protein content negatively, and vice versa. This phenomenon was in line with the seed trait changes during domestication from wild to cultivated soybeans. The identification of some special genes, such as *GmOLEO1* and *GmJAZ3*, has provided valuable targets for breeding soybean cultivars with larger seeds and higher protein content (Zhang et al. 2019; Hu et al. 2023a).

Abiotic stress and seed traits have also been reported to be correlated, according to some research. Abundant plant nutrients contribute to better survival in hazardous environments. For instance, lipid composition and content influence membrane stability and further regulate stress tolerance (Mikami and Murata 2003; Shi et al. 2008). Overexpression of *GmFAD3A* increases drought and salinity resistance, and silencing of this gene in soybean caused more sensitivity to drought and salinity stresses than occurred in the WT (Singh et al. 2022). The lipid regulator GmZF351 enhances stress resistance by directly activating the expression of the *GmCIPK9* and *GmSnRK* genes (Wei et al. 2023b). Compared with normal conditions, *GmZF351*-overexpressing soybeans produced a lower total FA content under salt stress conditions. GmNFYA could promote salt-responsive and oil-related gene expression in soybean (Lu et al. 2021a, b). Soybean seed oil, protein and other nutrients are also affected in response to abiotic stress (Wang and Frei 2011; Wijewardana et al. 2019; Ezzati Lotfabadi et al. 2022). An unfavorable environment usually causes accelerated leaf senescence and shortened seed development in plants (Black et al. 2000; Dupont and Altenbach 2003). Hence, the alteration of seed traits may be due to the influence of leaf photosynthesis and seed filling. However, other factors cannot be discounted.

## Conclusions and future perspectives

Soybean seed traits are a combination of a series of quantitative traits that are controlled, pleiotropically, by multiple genes. Although many genes have been verified and their molecular details were elucidated in *Arabidopsis* and rice, whether these genes function in a similar manner in soybean remains largely unknown. The complex soybean genome has been a main factor hindering the identification of functional genes. The size of the soybean genome is more than 1 Gb, and the majority of genes occur in multiple copies due to genome duplications (Schmutz et al. 2010; Shen et al. 2018a). As a diploidized tetraploid plant, soybean has significant functional redundancy, and knocking out some regulatory genes, singly, usually results in no obvious alteration in phenotypes, whereas overexpressing the corresponding genes may influence phenotypes significantly (Hu et al. 2023a; Wang et al. 2023). This functional redundancy may be because different genes produce the same metabolic outcome or possess overlapping molecular functions (Zhang et al. 2018b). The development of new technologies may contribute to overcoming this adversity within gene paralogs. For example, the transportome-scale artificial microRNA approach, which was designed to target a specific group of redundant members, has successfully identified ABCG transporters with functional redundancy in *Arabidopsis* (Zhang et al. 2021).

In addition, the soybean regulatory networks are not fully understood, perhaps owing to the limited number of genes whose functions have been confirmed in soybean by transgenic approaches. Seed traits vary in plants, even though the signaling pathways may be relatively conserved. Overexpression of several soybean genes, such as *GmJAZ3*, *GmZF351* and *GmZF392*, produce similar phenotypes in *Arabidopsis* and soybean, indicating that the corresponding genes may function similarly in different plant species (Li et al. 2017; Lu et al. 2021a; Hu et al. 2023a). Meanwhile, ectopic expression of *Sesamum indicum SiDGAT1* and *Arabidopsis AtSINA2* in soybean also was shown to promote higher oil content (Wang et al. 2014; Yang et al. 2023). Additionally, some orthologs of the same gene family play similar roles in soybean and other plants. The *CYP78A* gene is highly conserved in different plants, and its overexpression positively regulates seed size in *Arabidopsis*, rice, wheat and soybean (Fang et al. 2012; Ma et al. 2015; Zhao et al. 2016; Maeda et al. 2019). *GmJAZ3* and *GmPLATZ* have been identified as seed weight regulators in soybean, and their orthologs in *Arabidopsis* and rice have similar functions in regulating seed/grain development (Hu et al. 2023a, b). Hence, it is also beneficial to elucidate genetic regulatory networks for soybean seed traits based on studies in other plant species.

Although some soybean functional genes have been identified, their applications in breeding remain challenging. Knocking negative regulators out with CRISPR–Cas9 has proven to be an effective strategy for soybean molecular breeding to achieve favorable seed traits. For positive regulators, the creation of novel soybean germplasms may be enabled by the elevation of regulator gene expression through editing the promoter regions and by the increase of regulator mRNA stability by editing the UTR regions. The alternation of active sites in the regulatory proteins may also serve as a beneficial approach for functional studies.

Overall, even though a number of genes have been identified to affect seed traits, we still only have very limited knowledge about their modules and pathways (Fig. 1). Whether and how these genes form networks for efficient regulation requires further investigation (Fig. 1). Creating novel soybean germplasms with higher seed weight and yield, as well as with desirable nutrient compositions, is of great importance and should be pursued long term. Regarding the pleiotropic effects related to soybean seed traits, the identification of more genes in the regulatory pathways may enable these traits to be uncoupled and in turn may accelerate soybean breeding programs to produce plants with desirable traits. Further gene functional studies are expected to advance our understanding of the soybean signaling pathways for seed trait regulation and to provide molecular tools for targeted soybean breeding.

## Data Availability

Data sharing is not applicable to this article for the reason that no data was generated or analyzed during the current study.

## References

[CR1] Al Amin N, Ahmad N, Wu N, Pu X, Ma T, Du Y, Bo X, Wang N, Sharif R, Wang P (2019). CRISPR-Cas9 mediated targeted disruption of FAD2–2 microsomal omega-6 desaturase in soybean (*Glycine max* L.). BMC Biotechnol.

[CR2] Bandillo N, Jarquin D, Song Q, Nelson R, Cregan P, Specht J, Lorenz A (2015). A population structure and genome-wide association analysis on the USDA soybean germplasm collection. Plant Genome.

[CR3] Bates PD, Stymne S, Ohlrogge J (2013). Biochemical pathways in seed oil synthesis. Curr Opin Plant Biol.

[CR4] Baud S, Wuilleme S, Lemoine R, Kronenberger J, Caboche M, Lepiniec L, Rochat C (2005). The AtSUC5 sucrose transporter specifically expressed in the endosperm is involved in early seed development in Arabidopsis. Plant J.

[CR5] Beilinson V, Chen Z, Shoemaker C, Fischer L, Goldberg B, Nielsen C (2002). Genomic organization of glycinin genes in soybean. Theor Appl Genet.

[CR6] Bian XH, Li W, Niu CF, Wei W, Hu Y, Han JQ, Lu X, Tao JJ, Jin M, Qin H (2020). A class B heat shock factor selected for during soybean domestication contributes to salt tolerance by promoting flavonoid biosynthesis. New Phytol.

[CR7] Black VJ, Black CR, Roberts JA, Stewart CA (2000). Impact of ozone on the reproductive development of plants. New Phytol.

[CR8] Cai Z, Xian P, Cheng Y, Zhong Y, Yang Y, Zhou Q, Lian T, Ma Q, Nian H, Ge L (2023). MOTHER-OF-FT-AND-TFL1 regulates the seed oil and protein content in soybean. New Phytol.

[CR9] Catsimpoolas N (1969). Isolation of glycinin subunits by isoelectric focusing in urea-mercaptoethanol. FEBS Lett.

[CR10] Chaudhury AM, Koltunow A, Payne T, Luo M, Tucker MR, Dennis ES, Peacock WJ (2001). Control of early seed development. Annu Rev Cell Dev Biol.

[CR11] Chen L, Zheng Y, Dong Z, Meng F, Sun X, Fan X, Zhang Y, Wang M, Wang S (2018). Soybean (*Glycine max*) WRINKLED1 transcription factor, GmWRI1a, positively regulates seed oil accumulation. Mol Genet Genomics.

[CR12] Chen X, Liu C, Guo P, Hao X, Pan Y, Zhang K, Liu W, Zhao L, Luo W, He J (2023). Differential SW16.1 allelic effects and genetic backgrounds contributed to increased seed weight after soybean domestication. J Integr Plant Biol.

[CR13] Déjardin A, Rochat C, Maugenest S, Boutin JP (1997). Purification, characterization and physiological role of sucrose synthase in the pea seed coat (*Pisum sativum* L.). Planta.

[CR14] Demorest ZL, Coffman A, Baltes NJ, Stoddard TJ, Clasen BM, Luo S, Retterath A, Yabandith A, Gamo ME, Bissen J (2016). Direct stacking of sequence-specific nuclease-induced mutations to produce high oleic and low linolenic soybean oil. BMC Plant Biol.

[CR15] Doebley JF, Gaut BS, Smith BD (2006). The molecular genetics of crop domestication. Cell.

[CR16] Du J, Wang S, He C, Zhou B, Ruan YL, Shou H (2017). Identification of regulatory networks and hub genes controlling soybean seed set and size using RNA sequencing analysis. J Exp Bot.

[CR17] Duan P, Rao Y, Zeng D, Yang Y, Xu R, Zhang B, Dong G, Qian Q, Li Y (2014). SMALL GRAIN 1, which encodes a mitogen-activated protein kinase kinase 4, influences grain size in rice. Plant J.

[CR18] Duan Z, Zhang M, Zhang Z, Liang S, Fan L, Yang X, Yuan Y, Pan Y, Zhou G, Liu S (2022). Natural allelic variation of GmST05 controlling seed size and quality in soybean. Plant Biotechnol J.

[CR19] Duan Z, Li Q, Wang H, He X, Zhang M (2023). Genetic regulatory networks of soybean seed size, oil and protein contents. Front Plant Sci.

[CR20] Dupont FM, Altenbach SB (2003). Molecular and biochemical impacts of environmental factors on wheat grain development and protein synthesis. J Cereal Sci.

[CR21] Ezzati Lotfabadi Z, Weisany W, Abdul-Razzak Tahir N, Mohammadi Torkashvand A (2022). Arbuscular mycorrhizal fungi species improve the fatty acids profile and nutrients status of soybean cultivars grown under drought stress. J Appl Microbiol.

[CR22] Fan J, Yan C, Xu C (2013). Phospholipid:diacylglycerol acyltransferase-mediated triacylglycerol biosynthesis is crucial for protection against fatty acid-induced cell death in growing tissues of Arabidopsis. Plant J.

[CR23] Fang W, Wang Z, Cui R, Li J, Li Y (2012). Maternal control of seed size by EOD3/CYP78A6 in Arabidopsis thaliana. Plant J.

[CR24] Figueiredo DD, Kohler C (2014). Signalling events regulating seed coat development. Biochem Soc Trans.

[CR25] Flores T, Karpova O, Su X, Zeng P, Bilyeu K, Sleper DA, Nguyen HT, Zhang ZJ (2008). Silencing of GmFAD3 gene by siRNA leads to low alpha-linolenic acids (18:3) of fad3-mutant phenotype in soybean [*Glycine max* (Merr.)]. Transgenic Res.

[CR26] Gaut BS, Seymour DK, Liu Q, Zhou Y (2018). Demography and its effects on genomic variation in crop domestication. Nat Plants.

[CR27] Ge L, Yu J, Wang H, Luth D, Bai G, Wang K, Chen R (2016). Increasing seed size and quality by manipulating BIG SEEDS1 in legume species. Proc Natl Acad Sci USA.

[CR28] Goettel W, Zhang H, Li Y, Qiao Z, Jiang H, Hou D, Song Q, Pantalone VR, Song BH, Yu D (2022). POWR1 is a domestication gene pleiotropically regulating seed quality and yield in soybean. Nat Commun.

[CR29] Goldberg RB, Barker SJ, Perez-Grau L (1989). Regulation of gene expression during plant embryogenesis. Cell.

[CR30] Graham IA (2008). Seed storage oil mobilization. Annu Rev Plant Biol.

[CR31] Gu Y, Li W, Jiang H, Wang Y, Gao H, Liu M, Chen Q, Lai Y, He C (2017). Differential expression of a WRKY gene between wild and cultivated soybeans correlates to seed size. J Exp Bot.

[CR32] Guo W, Chen L, Chen H, Yang H, You Q, Bao A, Chen S, Hao Q, Huang Y, Qiu D (2020). Overexpression of GmWRI1b in soybean stably improves plant architecture and associated yield parameters, and increases total seed oil production under field conditions. Plant Biotechnol J.

[CR33] Harada JJ, Barker SJ, Goldberg RB (1989). Soybean beta-conglycinin genes are clustered in several DNA regions and are regulated by transcriptional and posttranscriptional processes. Plant Cell.

[CR34] Harwood JL (1988). Fatty acid metabolism. Ann Rev Plant Physiol Plant Mol Biol.

[CR35] Haun W, Coffman A, Clasen BM, Demorest ZL, Lowy A, Ray E, Retterath A, Stoddard T, Juillerat A, Cedrone F (2014). Improved soybean oil quality by targeted mutagenesis of the fatty acid desaturase 2 gene family. Plant Biotechnol J.

[CR36] Hoshino T, Watanabe S, Takagi Y, Anai T (2014). A novel GmFAD3-2a mutant allele developed through TILLING reduces alpha-linolenic acid content in soybean seed oil. Breed Sci.

[CR37] Hu D, Li X, Yang Z, Liu S, Hao D, Chao M, Zhang J, Yang H, Su X, Jiang M (2022). Downregulation of a gibberellin 3β-hydroxylase enhances photosynthesis and increases seed yield in soybean. New Phytol.

[CR38] Hu Y, Liu Y, Tao JJ, Lu L, Jiang ZH, Wei JJ, Wu CM, Yin CC, Li W, Bi YD (2023). GmJAZ3 interacts with GmRR18a and GmMYC2a to regulate seed traits in soybean. J Integr Plant Biol.

[CR39] Hu Y, Liu Y, Lu L, Tao JJ, Cheng T, Jin M, Wang ZY, Wei JJ, Jiang ZH, Sun WC (2023). Global analysis of seed transcriptomes reveals a novel PLATZ regulator for seed size and weight control in soybean. New Phytol.

[CR40] Huang K, Wang D, Duan P, Zhang B, Xu R, Li N, Li Y (2017). WIDE AND THICK GRAIN 1, which encodes an otubain-like protease with deubiquitination activity, influences grain size and shape in rice. Plant J.

[CR41] Hyten DL, Song Q, Zhu Y, Choi IY, Nelson RL, Costa JM, Specht JE, Shoemaker RC, Cregan PB (2006). Impacts of genetic bottlenecks on soybean genome diversity. Proc Natl Acad Sci USA.

[CR42] Jing G, Tang D, Yao Y, Su Y, Shen Y, Bai Y, Jing W, Zhang Q, Lin F, Guo D (2021). Seed specifically over-expressing DGAT2A enhances oil and linoleic acid contents in soybean seeds. Biochem Biophys Res Commun.

[CR43] Jo L, Pelletier JM, Hsu SW, Baden R, Goldberg RB, Harada JJ (2020). Combinatorial interactions of the LEC1 transcription factor specify diverse developmental programs during soybean seed development. Proc Natl Acad Sci USA.

[CR44] Jolivet P, Roux E, D'Andrea S, Davanture M, Negroni L, Zivy M, Chardot T (2004). Protein composition of oil bodies in *Arabidopsis thaliana* ecotype WS. Plant Physiol Biochem.

[CR45] Kanai M, Yamada T, Hayashi M, Mano S, Nishimura M (2019). Soybean (*Glycine max* L.) triacylglycerol lipase GmSDP1 regulates the quality and quantity of seed oil. Sci Rep.

[CR46] Kennedy EP (1961). Biosynthesis of complex lipids. Fed Proc.

[CR47] Kim MY, Lee S, Van K, Kim TH, Jeong SC, Choi IY, Kim DS, Lee YS, Park D, Ma J (2010). Whole-genome sequencing and intensive analysis of the undomesticated soybean (Glycine soja Sieb. and Zucc.) genome. Proc Natl Acad Sci U S A.

[CR48] Kinsella JE (1979). Functional-properties of soy proteins. J Am Oil Chem Soc.

[CR49] Komatsu S, Hirano H (1991). Plant basic 7 S globulin-like proteins have insulin and insulin-like growth factor binding activity. FEBS Lett.

[CR50] Komatsu S, Wang X, Yin X, Nanjo Y, Ohyanagi H, Sakata K (2017). Integration of gel-based and gel-free proteomic data for functional analysis of proteins through Soybean Proteome Database. J Proteomics.

[CR51] Lakhssassi N, Zhou Z, Liu S, Colantonio V, AbuGhazaleh A, Meksem K (2017). Characterization of the FAD2 gene family in soybean reveals the limitations of gel-based TILLING in genes with high copy number. Front Plant Sci.

[CR52] Lakhssassi N, Zhou Z, Liu S, Piya S, Cullen MA, El Baze A, Knizia D, Patil GB, Badad O, Embaby MG (2020). Soybean TILLING-by-Sequencing^+^ reveals the role of novel GmSACPD members in unsaturated fatty acid biosynthesis while maintaining healthy nodules. J Exp Bot.

[CR53] Le BH, Wagmaister JA, Kawashima T, Bui AQ, Harada JJ, Goldberg RB (2007). Using genomics to study legume seed development. Plant Physiol.

[CR54] Li N, Li Y (2015). Maternal control of seed size in plants. J Exp Bot.

[CR55] Li YH, Zhou G, Ma J, Jiang W, Jin LG, Zhang Z, Guo Y, Zhang J, Sui Y, Zheng L (2014). De novo assembly of soybean wild relatives for pan-genome analysis of diversity and agronomic traits. Nat Biotechnol.

[CR56] Li QT, Lu X, Song QX, Chen HW, Wei W, Tao JJ, Bian XH, Shen M, Ma B, Zhang WK (2017). Selection for a zinc-finger protein contributes to seed oil increase during soybean domestication. Plant Physiol.

[CR57] Li N, Xu R, Duan P, Li Y (2018). Control of grain size in rice. Plant Reprod.

[CR58] Li N, Xu R, Li Y (2019). Molecular networks of seed size control in plants. Annu Rev Plant Biol.

[CR59] Li C, Li YH, Li Y, Lu H, Hong H, Tian Y, Li H, Zhao T, Zhou X, Liu J (2020). A domestication-associated gene GmPRR3b regulates the circadian clock and flowering time in soybean. Mol Plant.

[CR60] Li J, Zhang Y, Ma R, Huang W, Hou J, Fang C, Wang L, Yuan Z, Sun Q, Dong X (2022). Identification of ST1 reveals a selection involving hitchhiking of seed morphology and oil content during soybean domestication. Plant Biotechnol J.

[CR61] Li H, Zhou R, Liu P, Yang M, Xin D, Liu C, Zhang Z, Wu X, Chen Q, Zhao Y (2023). Design of high-monounsaturated fatty acid soybean seed oil using GmPDCTs knockout via a CRISPR-Cas9 system. Plant Biotechnol J.

[CR62] Liang Q, Chen L, Yang X, Yang H, Liu S, Kou K, Fan L, Zhang Z, Duan Z, Yuan Y (2022). Natural variation of Dt2 determines branching in soybean. Nat Commun.

[CR63] Liu B, Watanabe S, Uchiyama T, Kong F, Kanazawa A, Xia Z, Nagamatsu A, Arai M, Yamada T, Kitamura K (2010). The soybean stem growth habit gene Dt1 is an ortholog of Arabidopsis TERMINAL FLOWER1. Plant Physiol.

[CR64] Liu YF, Li QT, Lu X, Song QX, Lam SM, Zhang WK, Ma B, Lin Q, Man WQ, Du WG (2014). Soybean GmMYB73 promotes lipid accumulation in transgenic plants. BMC Plant Biol.

[CR65] Liu S, Hua L, Dong S, Chen H, Zhu X, Jiang J, Zhang F, Li Y, Fang X, Chen F (2015). OsMAPK6, a mitogen-activated protein kinase, influences rice grain size and biomass production. Plant J.

[CR66] Liu JY, Zhang YW, Han X, Zuo JF, Zhang Z, Shang H, Song Q, Zhang YM (2020). An evolutionary population structure model reveals pleiotropic effects of GmPDAT for traits related to seed size and oil content in soybean. J Exp Bot.

[CR67] Liu S, Zhang M, Feng F, Tian Z (2020). Toward a "Green Revolution" for soybean. Mol Plant.

[CR68] Liu Y, Du H, Li P, Shen Y, Peng H, Liu S, Zhou GA, Zhang H, Liu Z, Shi M (2020). Pan-genome of wild and cultivated soybeans. Cell.

[CR69] Lu X, Li QT, Xiong Q, Li W, Bi YD, Lai YC, Liu XL, Man WQ, Zhang WK, Ma B (2016). The transcriptomic signature of developing soybean seeds reveals the genetic basis of seed trait adaptation during domestication. Plant J.

[CR70] Lu S, Zhao X, Hu Y, Liu S, Nan H, Li X, Fang C, Cao D, Shi X, Kong L (2017). Natural variation at the soybean J locus improves adaptation to the tropics and enhances yield. Nat Genet.

[CR71] Lu X, Xiong Q, Cheng T, Li QT, Liu XL, Bi YD, Li W, Zhang WK, Ma B, Lai YC (2017). A PP2C-1 allele underlying a quantitative trait locus enhances soybean 100-seed weight. Mol Plant.

[CR72] Lu L, Wei W, Li QT, Bian XH, Lu X, Hu Y, Cheng T, Wang ZY, Jin M, Tao JJ (2021). A transcriptional regulatory module controls lipid accumulation in soybean. New Phytol.

[CR73] Lu L, Wei W, Tao JJ, Lu X, Bian XH, Hu Y, Cheng T, Yin CC, Zhang WK, Chen SY (2021). Nuclear factor Y subunit GmNFYA competes with GmHDA13 for interaction with GmFVE to positively regulate salt tolerance in soybean. Plant Biotechnol J.

[CR74] Ma M, Wang Q, Li Z, Cheng H, Li Z, Liu X, Song W, Appels R, Zhao H (2015). Expression of TaCYP78A3, a gene encoding cytochrome P450 CYP78A3 protein in wheat (*Triticum aestivum* L.), affects seed size. Plant J.

[CR75] Ma J, Sun S, Whelan J, Shou H (2021). CRISPR/Cas9-mediated knockout of GmFATB1 significantly reduced the amount of saturated fatty acids in soybean seeds. Int J Mol Sci.

[CR76] Machado FB, Moharana KC, Almeida-Silva F, Gazara RK, Pedrosa-Silva F, Coelho FS, Grativol C, Venancio TM (2020). Systematic analysis of 1298 RNA-Seq samples and construction of a comprehensive soybean (*Glycine max*) expression atlas. Plant J.

[CR77] Maeda S, Dubouzet JG, Kondou Y, Jikumaru Y, Seo S, Oda K, Matsui M, Hirochika H, Mori M (2019). The rice CYP78A gene BSR2 confers resistance to *Rhizoctonia solani* and affects seed size and growth in Arabidopsis and rice. Sci Rep.

[CR78] Manan S, Ahmad MZ, Zhang G, Chen B, Haq BU, Yang J, Zhao J (2017). Soybean LEC2 regulates subsets of genes involved in controlling the biosynthesis and catabolism of seed storage substances and seed development. Front Plant Sci.

[CR79] Miao L, Yang S, Zhang K, He J, Wu C, Ren Y, Gai J, Li Y (2020). Natural variation and selection in GmSWEET39 affect soybean seed oil content. New Phytol.

[CR80] Mikami K, Murata N (2003). Membrane fluidity and the perception of environmental signals in cyanobacteria and plants. Prog Lipid Res.

[CR81] Moles AT, Ackerly DD, Webb CO, Tweddle JC, Dickie JB, Westoby M (2005). A brief history of seed size. Science.

[CR82] Napier JA, Stobart AK, Shewry PR (1996). The structure and biogenesis of plant oil bodies: the role of the ER membrane and the oleosin class of proteins. Plant Mol Biol.

[CR83] Nguyen QT, Kisiala A, Andreas P, Neil Emery RJ, Narine S (2016). Soybean seed development: fatty acid and phytohormone metabolism and their interactions. Curr Genomics.

[CR84] Nguyen CX, Paddock KJ, Zhang Z, Stacey MG (2020). GmKIX8-1 regulates organ size in soybean and is the causative gene for the major seed weight QTL qSw17-1. New Phytol.

[CR85] Nielsen NC (1985). The structure and complexity of the 11S polypeptides in soybeans. J Am Oil Chem Soc.

[CR86] Nielsen NC, Dickinson CD, Cho TJ, Thanh VH, Scallon BJ, Fischer RL, Sims TL, Drews GN, Goldberg RB (1989). Characterization of the glycinin gene family in soybean. Plant Cell.

[CR87] Ohlrogge J, Browse J (1995). Lipid biosynthesis. Plant Cell.

[CR88] Okuley J, Lightner J, Feldmann K, Yadav N, Lark E, Browse J (1994). Arabidopsis FAD2 gene encodes the enzyme that is essential for polyunsaturated lipid synthesis. Plant Cell.

[CR89] Rawsthorne S (2002). Carbon flux and fatty acid synthesis in plants. Prog Lipid Res.

[CR90] Sasaki Y, Nagano Y (2004). Plant acetyl-CoA carboxylase: structure, biosynthesis, regulation, and gene manipulation for plant breeding. Biosci Biotechnol Biochem.

[CR91] Schmutz J, Cannon SB, Schlueter J, Ma J, Mitros T, Nelson W, Hyten DL, Song Q, Thelen JJ, Cheng J (2010). Genome sequence of the palaeopolyploid soybean. Nature.

[CR92] Settlage SB, Kwanyuen P, Wilson RF (1998). Relation between diacylglycerol acyltransferase activity and oil concentration in soybean. J Am Oil Chem Soc.

[CR93] Shen Y, Liu J, Geng H, Zhang J, Liu Y, Zhang H, Xing S, Du J, Ma S, Tian Z (2018). De novo assembly of a Chinese soybean genome. Sci China Life Sci.

[CR94] Shen Y, Zhang J, Liu Y, Liu S, Liu Z, Duan Z, Wang Z, Zhu B, Guo YL, Tian Z (2018). DNA methylation footprints during soybean domestication and improvement. Genome Biol.

[CR95] Shen Y, Du H, Liu Y, Ni L, Wang Z, Liang C, Tian Z (2019). Update soybean Zhonghuang 13 genome to a golden reference. Sci China Life Sci.

[CR96] Shi Y, An L, Zhang M, Huang C, Zhang H, Xu S (2008). Regulation of the plasma membrane during exposure to low temperatures in suspension-cultured cells from a cryophyte (*Chorispora bungeana*). Protoplasma.

[CR97] Silva E, Belinato JR, Porto C, Nunes E, Guimarães F, Meyer MC, Pilau EJ (2021). Soybean metabolomics based in mass spectrometry: decoding the plant's signaling and defense responses under biotic stress. J Agric Food Chem.

[CR98] Singh AK, Fu DQ, El-Habbak M, Navarre D, Ghabrial S, Kachroo A (2011). Silencing genes encoding omega-3 fatty acid desaturase alters seed size and accumulation of Bean pod mottle virus in soybean. Mol Plant Microbe Interact.

[CR99] Singh A, Meena M, Kumar D, Dubey AK, Hassan MI (2015). Structural and functional analysis of various globulin proteins from soy seed. Crit Rev Food Sci Nutr.

[CR100] Singh AK, Raina SK, Kumar M, Aher L, Ratnaparkhe MB, Rane J, Kachroo A (2022). Modulation of GmFAD3 expression alters abiotic stress responses in soybean. Plant Mol Biol.

[CR101] Song XJ, Huang W, Shi M, Zhu MZ, Lin HX (2007). A QTL for rice grain width and weight encodes a previously unknown RING-type E3 ubiquitin ligase. Nat Genet.

[CR102] Song QX, Li QT, Liu YF, Zhang FX, Ma B, Zhang WK, Man WQ, Du WG, Wang GD, Chen SY (2013). Soybean GmbZIP123 gene enhances lipid content in the seeds of transgenic Arabidopsis plants. J Exp Bot.

[CR103] Staswick PE, Hermodson MA, Nielsen NC (1981). Identification of the acidic and basic subunit complexes of glycinin. J Biol Chem.

[CR104] Tang X, Su T, Han M, Wei L, Wang W, Yu Z, Xue Y, Wei H, Du Y, Greiner S (2017). Suppression of extracellular invertase inhibitor gene expression improves seed weight in soybean (*Glycine max*). J Exp Bot.

[CR105] Tang K, Yang S, Feng X, Wu T, Leng J, Zhou H, Zhang Y, Yu H, Gao J, Ma J (2020). GmNAP1 is essential for trichome and leaf epidermal cell development in soybean. Plant Mol Biol.

[CR106] Thanh VH, Shibasaki K (1977). Beta-conglycinin from soybean proteins. Isolation and immunological and physicochemical properties of the monomeric forms. Biochim Biophys Acta.

[CR107] Tian Z, Wang X, Lee R, Li Y, Specht JE, Nelson RL, McClean PE, Qiu L, Ma J (2010). Artificial selection for determinate growth habit in soybean. Proc Natl Acad Sci USA.

[CR108] Tsubokura Y, Hajika M, Kanamori H, Xia Z, Watanabe S, Kaga A, Katayose Y, Ishimoto M, Harada K (2012). The β-conglycinin deficiency in wild soybean is associated with the tail-to-tail inverted repeat of the a-subunit genes. Plant Mol Biol.

[CR109] Tzen J, Cao Y, Laurent P, Ratnayake C, Huang A (1993). Lipids, proteins, and structure of seed oil bodies from diverse species. Plant Physiol.

[CR110] Wang Y, Frei M (2011). Stressed food - the impact of abiotic environmental stresses on crop quality. Agr Ecosyst Environ.

[CR111] Wang HW, Zhang B, Hao YJ, Huang J, Tian AG, Liao Y, Zhang JS, Chen SY (2007). The soybean Dof-type transcription factor genes, GmDof4 and GmDof11, enhance lipid content in the seeds of transgenic Arabidopsis plants. Plant J.

[CR112] Wang Z, Huang W, Chang J, Sebastian A, Li Y, Li H, Wu X, Zhang B, Meng F, Li W (2014). Overexpression of SiDGAT1, a gene encoding acyl-CoA:diacylglycerol acyltransferase from *Sesamum indicum* L. increases oil content in transgenic Arabidopsis and soybean. Plant Cell Tiss Organ Cult.

[CR113] Wang M, Li W, Fang C, Xu F, Liu Y, Wang Z, Yang R, Zhang M, Liu S, Lu S (2018). Parallel selection on a dormancy gene during domestication of crops from multiple families. Nat Genet.

[CR114] Wang S, Liu S, Wang J, Yokosho K, Zhou B, Yu Y-C, Liu Z, Frommer WB, Ma JF, Chen L-Q (2020). Simultaneous changes in seed size, oil content and protein content driven by selection of SWEET homologues during soybean domestication. Natl Sci Rev.

[CR115] Wang Y, Yuan L, Su T, Wang Q, Gao Y, Zhang S, Jia Q, Yu G, Fu Y, Cheng Q (2020). Light- and temperature-entrainable circadian clock in soybean development. Plant Cell Environ.

[CR116] Wang F, Sun X, Liu B, Kong F, Pan X, Zhang H (2022). A polygalacturonase gene PG031 regulates seed coat permeability with a pleiotropic effect on seed weight in soybean. Theor Appl Genet.

[CR117] Wang Z, Wang Y, Shang P, Yang C, Yang M, Huang J, Ren B, Zuo Z, Zhang Q, Li W (2022). Overexpression of soybean GmWRI1a stably increases the seed oil content in soybean. Int J Mol Sci.

[CR118] Wang L, Yang Y, Yang Z, Li W, Hu D, Yu H, Li X, Cheng H, Kan G, Che Z (2023). GmFtsH25 overexpression increases soybean seed yield by enhancing photosynthesis and photosynthates. J Integr Plant Biol.

[CR119] Wei SM, Yong B, Jiang BW, An ZH, Wang Y, Li BB, Yang C, Zhu WW, Chen QS, He CY (2023). A loss-of-function mutant allele of a glycosyl hydrolase gene has been co-opted for seed weight control during soybean domestication. J Integr Plant Biol.

[CR120] Wei W, Lu L, Bian XH, Li QT, Han JQ, Tao JJ, Yin CC, Lai YC, Li W, Bi YD (2023). Zinc-finger protein GmZF351 improves both salt and drought stress tolerance in soybean. J Integr Plant Biol.

[CR121] Westoby M, Leishman M, Lord J (1996). Comparative ecology of seed size and dispersal. Phil Trans R Soc Lond B.

[CR122] Wijewardana C, Reddy KR, Bellaloui N (2019). Soybean seed physiology, quality, and chemical composition under soil moisture stress. Food Chem.

[CR123] Wu N, Lu Q, Wang P, Zhang Q, Zhang J, Qu J, Wang N (2020). Construction and analysis of GmFAD2-1A and GmFAD2-2A soybean fatty acid desaturase mutants based on CRISPR/Cas9 technology. Int J Mol Sci.

[CR124] Xie M, Chung CY, Li MW, Wong FL, Wang X, Liu A, Wang Z, Leung AK, Wong TH, Tong SW (2019). A reference-grade wild soybean genome. Nat Commun.

[CR125] Xu R, Duan P, Yu H, Zhou Z, Zhang B, Wang R, Li J, Zhang G, Zhuang S, Lyu J (2018). Control of grain size and weight by the OsMKKK10-OsMKK4-OsMAPK6 signaling pathway in rice. Mol Plant.

[CR126] Xu Y, Yan F, Liu Y, Wang Y, Gao H, Zhao S, Zhu Y, Wang Q, Li J (2021). Quantitative proteomic and lipidomics analyses of high oil content GmDGAT1-2 transgenic soybean illustrate the regulatory mechanism of lipoxygenase and oleosin. Plant Cell Rep.

[CR127] Yadav NS, Wierzbicki A, Aegerter M, Caster CS, Pérez-Grau L, Kinney AJ, Hitz WD, Booth JR, Schweiger B, Stecca KL (1993). Cloning of higher plant omega-3 fatty acid desaturases. Plant Physiol.

[CR128] Yang J, Mao T, Geng Z, Xue W, Ma L, Jin Y, Guo P, Qiu Z, Wang L, Yu C (2023). Constitutive expression of AtSINA2 from Arabidopsis improves grain yield, seed oil and drought tolerance in transgenic soybean. Plant Physiol Biochem.

[CR129] Yi X, Liu J, Chen S, Wu H, Liu M, Xu Q, Lei L, Lee S, Zhang B, Kudrna D (2022). Genome assembly of the JD17 soybean provides a new reference genome for comparative genomics. G3 (bethesda).

[CR130] Yu B, He X, Tang Y, Chen Z, Zhou L, Li X, Zhang C, Huang X, Yang Y, Zhang W (2023). Photoperiod controls plant seed size in a CONSTANS-dependent manner. Nat Plants.

[CR131] Yue Y, Sun S, Li J, Yu H, Wu H, Sun B, Li T, Han T, Jiang B (2021). GmFULa improves soybean yield by enhancing carbon assimilation without altering flowering time or maturity. Plant Cell Rep.

[CR132] Zhang M, Fan J, Taylor DC, Ohlrogge JB (2009). DGAT1 and PDAT1 acyltransferases have overlapping functions in Arabidopsis triacylglycerol biosynthesis and are essential for normal pollen and seed development. Plant Cell.

[CR133] Zhang YQ, Lu X, Zhao FY, Li QT, Niu SL, Wei W, Zhang WK, Ma B, Chen SY, Zhang JS (2016). Soybean GmDREBL increases lipid content in seeds of transgenic Arabidopsis. Sci Rep.

[CR134] Zhang D, Sun L, Li S, Wang W, Ding Y, Swarm SA, Li L, Wang X, Tang X, Zhang Z (2018). Elevation of soybean seed oil content through selection for seed coat shininess. Nat Plants.

[CR135] Zhang Y, Nasser V, Pisanty O, Omary M, Wulff N, Di Donato M, Tal I, Hauser F, Hao P, Roth O (2018). A transportome-scale amiRNA-based screen identifies redundant roles of Arabidopsis ABCB6 and ABCB20 in auxin transport. Nat Commun.

[CR136] Zhang D, Zhang H, Hu Z, Chu S, Yu K, Lv L, Yang Y, Zhang X, Chen X, Kan G (2019). Artificial selection on GmOLEO1 contributes to the increase in seed oil during soybean domestication. PLoS Genet.

[CR137] Zhang Y, Kilambi HV, Liu J, Bar H, Lazary S, Egbaria A, Ripper D, Charrier L, Belew ZM, Wulff N (2021). ABA homeostasis and long-distance translocation are redundantly regulated by ABCG ABA importers. Science Adv.

[CR138] Zhang M, Liu S, Wang Z, Yuan Y, Zhang Z, Liang Q, Yang X, Duan Z, Liu Y, Kong F (2022). Progress in soybean functional genomics over the past decade. Plant Biotechnol J.

[CR139] Zhao B, Dai A, Wei H, Yang S, Wang B, Jiang N, Feng X (2016). Arabidopsis KLU homologue GmCYP78A72 regulates seed size in soybean. Plant Mol Biol.

[CR140] Zhao Y, Cao P, Cui Y, Liu D, Li J, Zhao Y, Yang S, Zhang B, Zhou R, Sun M (2021). Enhanced production of seed oil with improved fatty acid composition by overexpressing NAD^+^-dependent glycerol-3-phosphate dehydrogenase in soybean. J Integr Plant Biol.

[CR141] Zhou Z, Jiang Y, Wang Z, Gou Z, Lyu J, Li W, Yu Y, Shu L, Zhao Y, Ma Y (2015). Resequencing 302 wild and cultivated accessions identifies genes related to domestication and improvement in soybean. Nat Biotechnol.

[CR142] Zhu W, Yang C, Yong B, Wang Y, Li B, Gu Y, Wei S, An Z, Sun W, Qiu L (2022). An enhancing effect attributed to a nonsynonymous mutation in SOYBEAN SEED SIZE 1, a SPINDLY-like gene, is exploited in soybean domestication and improvement. New Phytol.

